# MiR-33a suppresses breast cancer cell proliferation and metastasis by targeting ADAM9 and ROS1

**DOI:** 10.1007/s13238-015-0223-8

**Published:** 2015-10-27

**Authors:** Chuankai Zhang, Yunda Zhang, Weiji Ding, Yancheng Lin, Zhengjie Huang, Qi Luo

**Affiliations:** Department of Gastrointestinal Surgery, First Affiliated Hospital of Xiamen University, Xiamen, 361003 China; State Key Laboratory of Cellular Stress Biology, Innovation Center for Cell Signaling Network, School of Life Sciences, Xiamen University, Xiamen, 361102 China; Department of Gastrointestinal Surgery, First Clinical Medical College of Fujian Medical University, Fuzhou, 350005 China

**Keywords:** miR-33a, breast cancer, proliferation, metastasis

## Abstract

MicroRNAs (miRNAs) are small noncoding RNAs that have a pivotal role in the post-transcriptional regulation of gene expression by sequence-specifically targeting multiple mRNAs. Although miR-33a was recently reported to play an important role in lipid homeostasis, atherosclerosis, and hepatic fibrosis, the functions of miR-33a in tumor progression and metastasis are largely unknown. Here, we found that downregulated miR-33a in breast cancer tissues correlates with lymph node metastasis. MiR-33a expression is significantly lower in the highly metastatic breast cancer cell lines than the noncancerous breast epithelial cells and non-metastatic breast cancer cells. Moreover, the overexpression of miR-33a in metastatic breast cancer cells remarkably decreases cell proliferation and invasion *in vitro* and significantly inhibits tumor growth and lung metastasis *in vivo*, whereas its knockdown in non-metastatic breast cancer cells significantly enhances cell proliferation and invasion *in vitro* and promotes tumor growth and lung metastasis *in vivo*. Combining bioinformatics prediction and biochemical analyses, we showed that ADAM9 and ROS1 are direct downstream targets of miR-33a. These findings identified miR-33a as a negative regulator of breast cancer cell proliferation and metastasis.

## INTRODUCTION

Breast cancer is the leading cause of cancer-related death for women worldwide, and distant metastasis is the most common cause of death in breast cancer patients (Hong and Dong, 2014). Similar to other cancers, the invasion-metastasis cascade of breast cancer consists of multiple steps, including local invasion, entry into the circulation, arrival at distant secondary sites, extravasation and colonization in distant organs (Chaffer and Weinberg, 2011; Wan et al., 2013; Wang and Ouyang, 2012). Considerable progress in breast cancer treatment has been made over the past decades; however, metastatic breast cancer is still difficult to cure. The lack of curative treatment options for metastatic breast cancer patients emphasizes the need to better understand the molecular mechanisms that drive tumor metastasis.

MicroRNAs (miRNAs) are a class of endogenous small noncoding RNAs that are typically 18–22 nucleotides in length. By binding to completely or partially complementary sites in the 3′-untranslated-region (3′UTR) of target mRNAs, miRNAs suppress the protein translation of these transcripts and/or degrade target mRNAs. Currently, miRNAs have been shown to contribute to regulate tumor onset, growth, and progression (Aleckovic and Kang, 2015; Ruan et al., 2009; Volinia et al., 2012). A single miRNA can downregulate the expression of multiple target genes, thereby coordinately inhibiting or promoting tumor metastasis. Therefore, miRNAs may be attractive targets for modulating the invasion-metastasis cascade. MiR-33a is an intronic miRNA located in intronic sequences of the sterol-response-element-binding protein gene SREBF2, and it plays a critical role in the regulation of cholesterol and fatty acid metabolism (Marquart et al., 2010; Najafi-Shoushtari et al., 2010; Rayner et al., 2011; Rayner et al., 2010). MiR-33a is also involved in liver fibrosis aggravation in nonalcoholic steatohepatitis in mice (Li et al., 2014). Interestingly, a recent report demonstrated that higher miR-33a expression correlates with poor prognosis for GBM patients and promotes glioma-initiating cell self-renewal via the activation of the PKA and NOTCH pathways by targeting PDE8A and UVRAG (Wang et al., 2014). However, little is known about the function of miR-33a in breast cancer progression. In this report, we revealed that miR-33a is downregulated in breast cancer tissues. The expression level of miR-33a in breast cancer tissues is inversely correlated with lymph node metastasis. MiR-33a overexpression inhibits breast cancer cell proliferation and invasion *in vitro* and suppresses tumor growth and lung metastasis of breast cancer cells *in vivo*. Conversely, miR-33a knockdown significantly enhances breast cancer cell proliferation and invasion *in vitro* and promotes tumor growth and lung metastasis *in vivo*. Our study demonstrated that miR-33a plays a tumor suppressor role in breast cancer metastasis.

## RESULTS

### Identification of miR-33a as a tumor suppressor gene in breast cancer

**Figure 1:**
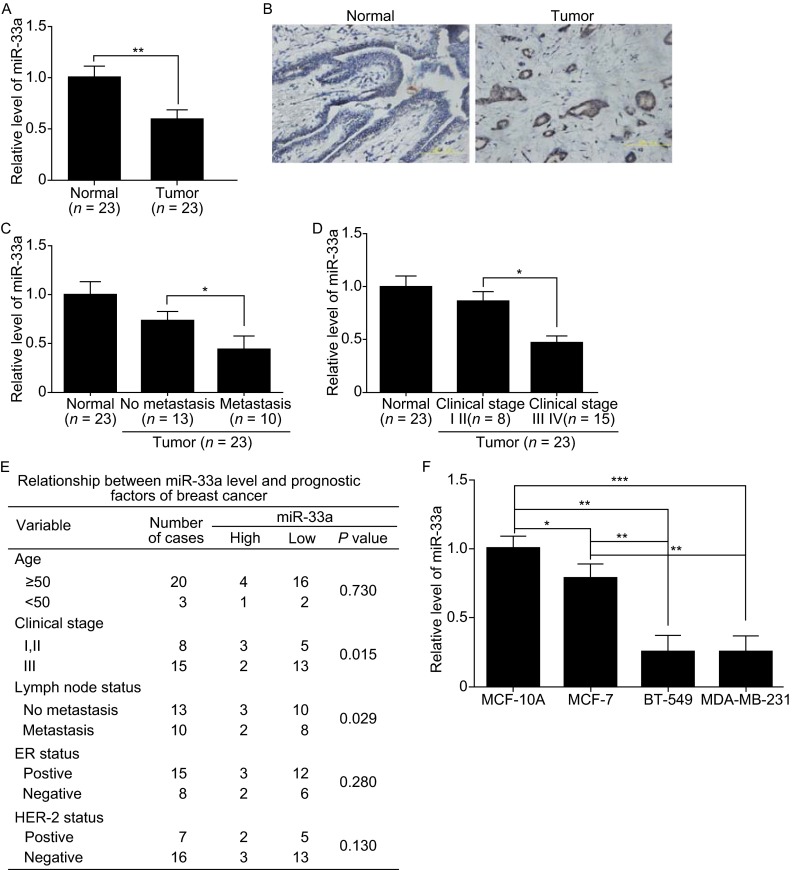
**MiR-33a is markedly downregulated in human breast cancer tissues and metastatic breast cancer cell lines**. (A) qRT-PCR analysis of miR-33a expression in human breast cancer tissue samples and their matched normal breast tissues from 23 breast cancer patients. (B) *In situ* hybridization analysis of miR-33a expression in human breast cancer tissues and matched normal tissues. (C) Correlation between miR-33a expression and the lymph node metastasis status of breast cancer. (D) Correlation between miR-33a expression and the progression of the clinical stage of breast cancer. (E) Correlation between clinicopathological features and miR-33a expression in 23 breast cancer tissues. (F) qRT-PCR analysis of miR-33a expression in noncancerous human mammary epithelial cells and breast cancer cell lines with different metastatic potential. Scale bars = 100 μm; *P < 0.05; **P < 0.01; ***P < 0.001

Recent studies demonstrate that miR-33a is highly expressed in osteosarcoma, enhances osteosarcoma cell resistance to cisplatin by targeting Twist1 (Zhou et al., 2014), and promotes glioma-initiating cell self-renewal in glioma (Wang et al., 2014). However, miR-33a acts as a tumor suppressor miRNA in colon cancer by directly downregulating of the oncogene Pim-1 (Ibrahim et al., 2011; Thomas et al., 2012). Currently, the function of miR-33a in breast cancer progression remains unknown. Microarray analysis by Blenkiron et al. showed that miR-33a expression is often lost in human primary breast cancers because of chromosome alterations, indicating that miR-33a may function as a tumor suppressor (Blenkiron et al., 2007). To determine the exact function of miR-33a in breast cancer, we performed real-time PCR and *in situ* hybridization assays to detect the expression level of miR-33a in breast cancer tissues and cell lines. As shown in Fig. 1A, in 23 cases matched breast cancer samples and normal breast tissues, miR-33a expression was significantly decreased in the breast cancer samples compared to the matched normal tissues. *In situ* hybridization assays confirmed that miR-33a was highly expressed in the normal breast tissue, whereas little signal was observed in tumor tissue (Fig. 1B). We further determined the correlation between the miR-33a level and the metastatic status of patients with breast cancer. We found that the expression of miR-33a was negatively associated with lymph node metastasis (Fig. 1C) and the progression of clinical stage (Fig. 1D) in breast cancer patients. The relevance between the miR-33a expression level and prognostic factors of breast cancer is summarized in Fig. 1E. We also observed that miR-33a expression was significantly lower in the highly metastatic breast cancer cell lines MDA-MB-231 and BT-549 than in the noncancerous breast epithelial cell line MCF-10A and non-metastatic breast cancer cell line MCF-7 (Fig. 1F). These results suggest that the miR-33a level is downregulated in breast cancer tissues and breast cancer cell lines and that it is negatively correlated with the metastatic ability of breast cancer cells.

### MiR-33a inhibits breast cancer cell growth, migration and invasion *in vitro*

**Figure 2:**
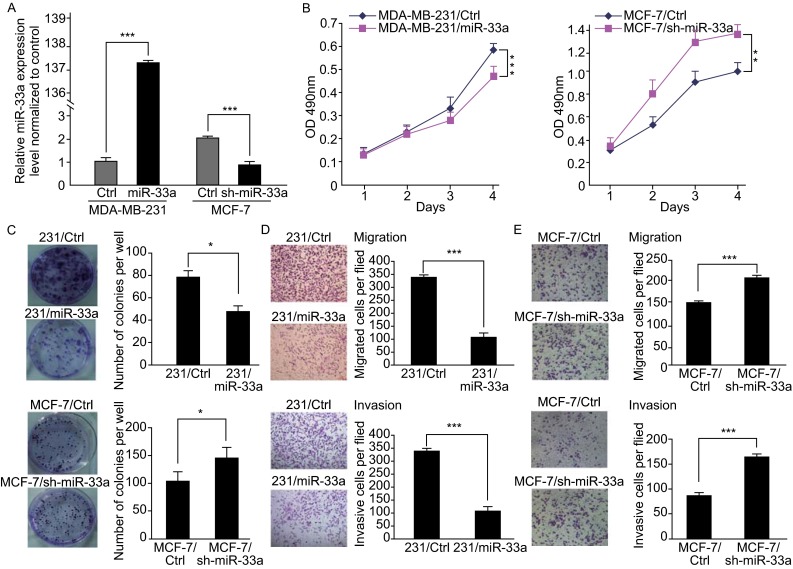
**MiR-33a inhibits breast cancer cell growth, migration, and invasion** ***in vitro***. (A) The miR-33a expression level in MDA-MB-231 cells after ectopic expression of miR-33a and the knockdown efficiency of miR-33a in MCF-7 cells were detected by real-time PCR; (B) The effect on cell proliferation of miR-33a overexpression in MDA-MB-231 cells or knockdown in MCF-7 cells was determined by the MTT assay; (C) Representative images show the colony formation of MDA-MB-231/miR-33a and MCF-7/sh-miR-33a and their control cells (left panel). Average colonies in each well for each group were counted from three independent experiments (right panel); (D) The effects of miR-33a overexpression in MDA-MB-231 cells on cell migration and invasion were analyzed by Transwell migration and Matrigel-coated Transwell invasion analyses; (E) The effects of miR-33a knockdown in MCF-7 cells on cell migration and invasion were analyzed by Transwell migration and Matrigel-coated Transwell invasion analyses. *P < 0.05; **P < 0.01; ***P < 0.001

To evaluate the biological function of miR-33a in breast cancer, the highly metastatic breast cancer cell line MDA-MB-231, which has very low endogenous miR-33a expression, was stably transfected with miR-33a by lentiviral infection, whereas endogenous miR-33a was knocked down in non-metastatic MCF-7 breast cancer cells via a lentivirus-based antagomir expression system (Fig. 2A). MTT assays showed that the ectopic overexpression of miR-33a inhibited the proliferation of MDA-MB-231 cells, whereas sh-miR-33a promoted MCF-7 cell proliferation (Fig. 2B). Moreover, colony formation assays revealed that miR-33a-overexpressing MDA-MB-231 cells displayed fewer colonies compared with the control group. Conversely, the knockdown of miR-33a in MCF-7 cells increased colony numbers (Fig. 2C). Because miR-33a expression was inversely correlated with the metastatic abilities of breast cancer cell lines, the effects of miR-33a on the migration and invasion of breast cancer cells were further examined by Transwell assays. As shown in Fig. 2D, miR-33a overexpression in MDA-MB-231 cells dramatically inhibited cell migration and invasion. In contrast, miR-33a knockdown in MCF-7 cells could promote cell migration and invasion (Fig. 2E). These results demonstrate that miR-33a can inhibit the proliferation, migration, and invasion of breast cancer cells *in vitro*.

### MiR-33a suppresses the tumor growth and lung metastasis of breast cancer cells *in vivo*

**Figure 3:**
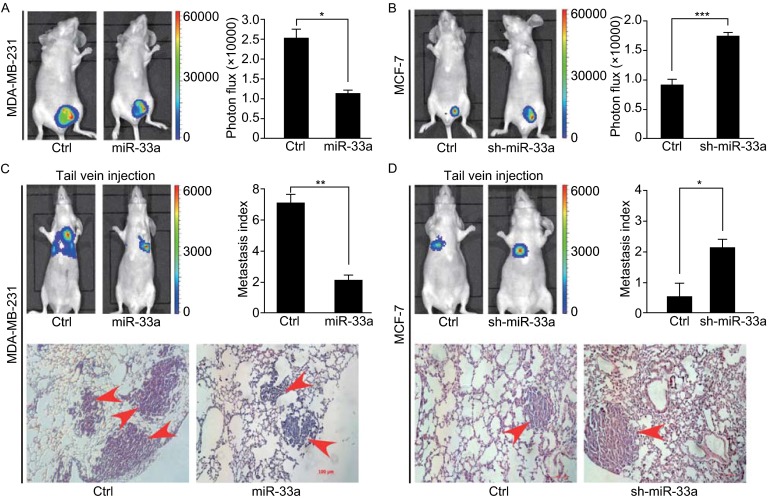
**MiR-33a suppresses tumor growth and lung metastasis of breast cancer cells** ***in vivo***. (A) Representative bioluminescence images of primary tumors in the nude mice orthotopically injected with MDA-MB-231/miR-33a or MDA-MB-231/ctrl cells in the mammary fat pad (left). Quantification of tumors in each group is shown (right); (B) Representative bioluminescence images of primary tumors in the nude mice orthotopically injected with MCF-7/sh-miR-33a or MCF-7/ctrl cells in the mammary fat pad (left). Quantification of tumors in each group is shown (right); (C) Representative bioluminescence images of mice injected with MDA-MB-231/miR-33a or MDA-MB-231/ctrl cells into tail vein to show lung metastases (top-left). Quantification of lung metastases was analyzed by bioluminescence measurement (top-right). Lung metastases in the mice were detected by H&E staining (bottom); (D) Representative bioluminescence images of mice injected with MCF-7/sh-miR-33a or MCF-7/ctrl cells into tail vein to show lung metastases (top-left). Quantification of lung metastases was analyzed by bioluminescence measurement (top-right). Lung metastases in the mice were detected by H&E staining (bottom). Scale bars = 100 μm; *P < 0.05; **P < 0.01; ***P < 0.001

To determine whether miR-33a can inhibit the tumor growth and metastasis of breast cancer cells *in vivo*, we generated luciferase-labeled MDA-MB-231/miR-33a cells, MCF-7/sh-miR-33a cells, and their control counterparts, and then injected them into the orthotopic sites or tail vein of nude mice. Thirty days after injection at orthotopic sites, the mice injected with MDA-MB-231/miR-33a cells displayed significant smaller and lower weight tumors compared with the mice injected with MDA-MB-231/ctrl cells (Fig. 3A), whereas the mice injected with MCF-7/sh-miR-33a cells formed larger tumors with higher weights than mice injected with MCF-7/ctrl cells (Fig. 3B). Moreover, bioluminescence imaging of mice injected with MDA-MB-231/miR-33a or MDA-MB-231/ctrl cells for 30 days showed that the lung metastasis of MDA-MB-231 cells was significantly impaired by the ectopic overexpression of miR-33a (Fig. 3C). However, mice bearing MCF-7/sh-miR-33a cells after tail vein injection for 42 days displayed large lung metastases compared with the control group (Fig. 3D). These data demonstrate that miR-33a suppresses the tumor growth and lung metastasis of breast cancer cells *in vivo*.

### ADAM9 and ROS1 are direct targets of miR-33a

**Figure 4:**
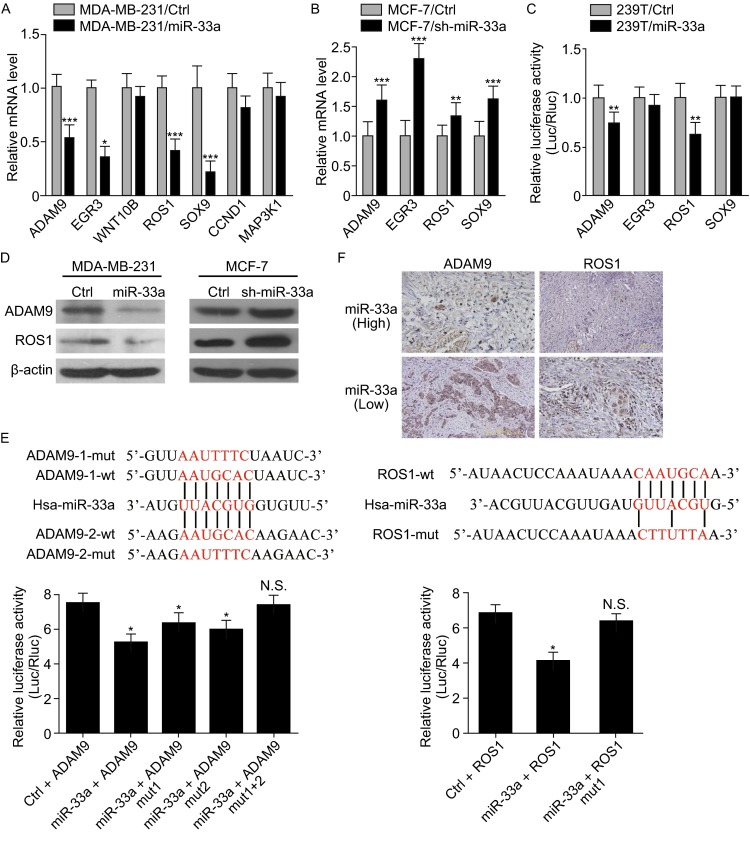
A**DAM9 and ROS1 are direct targets of miR-33a**. (A) The mRNA levels of predicted target genes of miR-33a in MDA-MB-231/miR-33a and MDA-MB-231/ctrl cells were analyzed by real-time PCR; (B) The mRNA levels of predicted target genes of miR-33a in MCF-7/sh-miR-33a and MCF-7/ctrl cells were further analyzed by real-time PCR; (C) The effects of miR-33a overexpression on the activity of the 3′UTRs of target genes in 293T cells were analyzed by the dual luciferase reporter assay; (D) The effect of miR-33a overexpression in MDA-MB-231 cells or knockdown in MCF-7 cells on the expression of ADAM9 and ROS1 was detected by Western blotting; (E) The effects of miR-33a expression on the activity of wild-type and mutant 3′UTRs of ADAM9 and ROS1 were analyzed by the dual luciferase reporter assay; (F) The levels of ADAM9 and ROS1 were negatively correlated with miR-33a expression in human breast cancer tissues. Scale bars = 200 μm; *P < 0.05; **P < 0.01; ***P < 0.001; N.S. no significance

To reveal the underlying mechanism by which miR-33a inhibits the tumor growth and lung metastasis of breast cancer cells, we used four *in silico* algorithms (Targetscan, miRanda, mirwalk, and Pictar) to predict the target genes of miR-33a and then used real-time PCR to detect the expression of putative miR-33a targets. We found four candidate targets with greater than 30% decreased expression upon ectopic miR-33a overexpression in MDA-MB-231 cells (Fig. 4A and 4B). To examine whether these four predicted oncogene targets were true targets of miR-33a, we constructed luciferase reporter vectors containing wild-type or mutant 3′UTRs of these candidate target genes. Luciferase activity assays revealed that miR-33a suppressed the expression of luciferase containing the 3′UTRs of ADAM9 and ROS1 compared with controls (Fig. 4C). We also performed Western blot analyses to examine the levels of ADAM9 and ROS1 proteins. As shown in Fig. 4D, the levels of ADAM9 and ROS1 were markedly decreased in MDA-MB-231/miR-33a cells compared with MDA-MB-231/ctrl cells. Conversely, the levels of ADAM9 and ROS1 were increased in MCF-7/sh-miR-33a cells compared with MCF-7/ctrl cells. We found two putative binding sites of miR-33a in the ADAM9 3′UTR and one putative binding site in the ROS1 3′UTR, and we then obliterated these miR-33a binding sites in the 3′UTRs of ADAM9 and ROS1 by QuickChange PCR (Zheng et al., 2004). As shown in Fig. 4E, the mutation of binding site 1, binding site 2, or both sites in the ADAM9 3′UTR reversed the miR-33a-induced downregulation of luciferase activity. Mutation of the binding sites of miR-33a in the ROS1 3′UTR also abrogated the suppressive effect of miR-33a overexpression. Immunohistochemical staining showed that breast cancer tissues with high miR-33a expression have low expression of ADAM9 and ROS1, whereas breast cancer tissues with low miR-33a expression exhibit high expression of ADAM9 and ROS1 (Fig. 4F). Taken together, these results indicate that ADAM9 and ROS1 are direct targets of miR-33a in breast cancer cells.

## DISCUSSION

Recent studies have revealed that the aberrant expression of miRNAs is involved in tumor progression; these miRNAs function by inhibiting their target genes, and they play critical roles in the coordination of tumor cell proliferation, invasion, intravasation, survival, extravasation, and/or colonization. Therefore, the identification of specific miRNAs and their targets involved in tumorigenesis and metastasis would provide important clues for identifying new diagnostic and therapeutic targets for cancer prevention and treatment. Interestingly, the microarray analysis by Blenkiron et al. revealed that miR-33a is often lost in human breast cancer (Blenkiron et al., 2007). Here, we found that miR-33a exhibits a decreased expression level in breast cancer tissues compared with matched normal tissues. Moreover, in patients with breast cancer, a correlation is observed between lower miR-33a expression and increased lymph node metastasis. MiR-33a inhibits breast cancer cell proliferation, migration, and invasion *in vitro* and suppresses tumor growth and lung metastasis *in vivo*. Therefore, our study identifies miR-33a as a tumor-suppressive gene in breast cancer metastasis.

Members of the ADAM family are involved in tumor metastasis (Rocks et al., 2008). One member of this family, ADAM9, is overexpressed in breast cancer (O’Shea et al., 2003). Silencing ADAM9 inhibits breast cancer cell invasion (Micocci et al., 2013). ROS1 is a newly identified receptor tyrosine kinase, and its chromosomal rearrangement results in constitutively active ROS1, which has potent transforming ability. ROS1 rearrangement can be used as a predictive marker for response to crizotinib, a tyrosine kinase inhibitor, in patients with advanced non-small cell lung cancer (NSCLC) (Bergethon et al., 2012; Shaw et al., 2014). Recently, a ROS1-rearranged NSCLC patient who had choroidal metastases that did not respond to initial chemotherapy but showed a rapid and complete response to crizotinib was reported (Lu et al., 2015). These reports demonstrate that ADAM9 and ROS1 are oncogenes that may contribute to tumor metastasis. In this report, using Targetscan, miRanda, miRwalk, and Pictar databases, we found that there are highly conserved miR-33a binding sites in the 3′UTRs of ADAM9 and ROS1. Moreover, the miR-33a level is inversely associated with the expression of ADAM9 and ROS1 in breast cancer tissues, indicating that miR-33a may inhibit breast cancer cell proliferation and metastasis, at least in part, by downregulating the levels of ADAM9 and ROS1.

Collectively, our study provides evidence that miR-33a is a novel tumor suppressor gene in breast cancer progression. MiR-33a expression is negatively correlated with lymph node metastasis in patients with breast cancer. MiR-33a might inhibit breast cancer cell proliferation and metastasis by suppressing ADAM9 and ROS1. Therefore, our findings provide a potential diagnostic and prognostic marker for breast cancer metastasis.

## MATERIALS AND METHODS

### Cell lines and clinical samples

MCF-7, MCF-10A, and 293T cells were obtained from Dr. Kunxin Luo (University of California at Berkeley, Berkeley, CA). MDA-MB-231 cells were provided by Dr. Guohong Hu (Institute of Health Sciences, Shanghai Institute for Biological Sciences, Shanghai). 293T cells were cultured in DMEM supplemented with 10% fetal bovine serum. MCF-7 and MDA-MB-231 cells were maintained in RPMI 1640 media supplemented with 10% fetal bovine serum.

Thirteen matched breast cancer, normal adjacent tissues, and lymph node metastases were collected from the First Affiliated Hospital of Xiamen University, Xiamen, China. Specimens were obtained with informed consent and the study was performed in accordance with the approved guidelines by the Ethics and Scientific Committees of Xiamen University.

### Plasmid construction and generation of stable cell lines

An hsa-miR-33a-containing flank region was amplified from human genomic DNA and inserted into pCDH-CMV-EF1-GFP+puro (System Biosciences). The entire lengths of the 3′UTRs of ADAM9 and ROS1 were cloned into the pMIR-REPORT miRNA Expression Reporter Vector (Ambion). To generate a miR-33a-expressing stable cell line, a lentivirus-mediated packaging system containing four plasmids, pCDH-miR-33a or control plasmid, pMDL, REV, and VSVG, was used. To stably knock down miR-33a in MCF-7 cells, pLL3.7-puro containing anti-miR-33a or anti-pre-miR-33a shRNA was co-transfected with pMDL, REV, and VSVG. The transfection and lentiviral infection processes were similar to those previously described (Fang et al., 2011).

### Western blotting

Western blotting was performed as described previously (Fang et al., 2011). Cell lysates were separated on SDS–polyacrylamide gels and immunoblot analysis was performed with primary antibodies against ADAM9 (Invitrogen), ROS1 (Cell Signaling), and β-actin (Millipore).

### Cell growth assay

For the MTT assay, cells were seeded and transfected in a 96-well plate. After transfection for 1, 2, 3, or 4 days, MTT was added to the cells, and the absorbance at 490 nm was measured. For colony formation assays, cells were seeded in 6-well plates and maintained in RPMI 1640 medium containing 10% FBS for 15 days. Colonies were fixed with 4% PFA, stained with 0.1% crystal violet for 15 min, and photographed using a digital camera. Experiments were repeated three times.

### Real-time quantitative PCR

Total RNA was extracted from the cultured cells or frozen tissues using TRIzol reagent (Invitrogen, Carlsbad, California, USA). For miRNA reverse transcription, cDNA was synthesized using TaqMan^®^ MicroRNA Reverse Transcription Kit (ABI) with 100 ng total RNA. The mRNA was reverse-transcribed by an RT-PCR kit (Invitrogen, Carlsbad, California, USA) according to the manufacturer’s instructions. Quantitative PCR was then performed with primers for miR-33a, ADAM9, EGR3, ROS1, SOX9, WNT10B, CCND1, and MAP3K1 using SYBR Green PCR Master Mix (Invitrogen, Carlsbad, California, USA) in a real-time PCR System (Applied Biosystems, Carlsbad, California, USA) following a standard quantitative PCR procedure. Primer sequences were as follows: miR-33a, 5′-GGGGGTGCATTGTAGTTG-3′ and 5′-TGCGTGTCGTGGAGTC-3′; ADAM9, 5′-GCTAGTTGGACTGGAGATTTGG-3′ and 5′-TTATTACCACAGGAGGGAGCAC-3′; EGR3, 5′-CTGCCTGACAATCTGTACCC-3′ and 5′-GTAGGTCACGGTCTTGTTGC-3′; ROS1, 5′-ATGGGCTCCTGTATTGGTTG-3′ and 5′-CATCAGTGCATTCTGGGAAA-3′; SOX9, 5′-GAGGAAGTCGGTGAAGAACG-3′ and 5′-GGAGTGCACCTCGCTCAT-3′; WNT10B, 5′-TGGGATGTGTAGCCTTCTCC-3′ and 5′-CCCAGCCAAAAGGAGTATGA-3′; CCND1, 5′-CCCTCGGTGCCTACTTCAA-3′ and 5′-CTCCTCGCACTTCTGTTCCT-3′; MAP3K1, 5′-TGATGTATGGAGTGTTGGCTG-3′ and 5′-AATGTGAAGGGATCGATGGAG-3′; GAPDH, 5′-GCACCGTCAAGGCTGAGAAC-3′ and 5′-TGGTGAAGACGCCAGTGGA-3′. Relative quantification was performed by normalization to the amount of GAPDH.

### MiRNA reporter luciferase assay

For luciferase reporter assays, 293T cells were seeded into a 24-well plate and co-transfected with 3′UTR-luciferase and either miR-33a or control plasmids. Cells were harvested after two days and assayed using the Dual-Glo Luciferase Assay System (Promega) to determine the relative luciferase activity. The luciferase activity was measured by a luciferin enzyme detection assay kit (Promega) and normalized to *Renilla* luciferase activity. Each treatment was performed in triplicate in three independent experiments.

### Cell motility and invasion assay

Migration and invasion assays were performed as described previously (Liu et al., 2014). All experiments were performed at least three times in triplicate.

### Animal studies

All experiments using animals were performed in accordance with a protocol approved by the Animal Care and Use Committee of Xiamen University. For orthotopic assays, 5 × 10^6^ MDA-MB-231 or MCF-7 cells in Matrigel (BD, Biosciences) plus growth media were injected into the mammary fat pads of nude mice (*n* = 3–4 per group). Mice were euthanized 30 days after orthotopic injection, and the growth of subcutaneous tumors was examined by live animal Lumina II system (Xenogen IVIS system). For tail vein metastasis, 1 × 10^6^ MDA-MB-231 cells or 3 × 10^6^ MCF-7 cells were injected into the tail veins of nude mice (*n* = 3 per group). Mice were euthanized 30 (MDA-MB-231) or 42 (MCF-7) days after tail vein injection, and the lung metastases were examined by live animal Lumina II system (Xenogen IVIS system) and further detected by H&E staining.

### Statistical analysis

All of our experiments were performed 3 biological repeats. All data were expressed as the mean ± SD. Statistical analysis was performed with Student’s *t* test. A *P* value less than 0.05 was considered statistically significant.

## References

[CR1] Aleckovic M, Kang Y (2015). Regulation of cancer metastasis by cell-free miRNAs. Biochim Biophys Acta.

[CR2] Bergethon K, Shaw AT, Ou SH, Katayama R, Lovly CM, McDonald NT, Massion PP, Siwak-Tapp C, Gonzalez A, Fang R (2012). ROS1 rearrangements define a unique molecular class of lung cancers. J Clin Oncol.

[CR3] Blenkiron C, Goldstein LD, Thorne NP, Spiteri I, Chin SF, Dunning MJ, Barbosa-Morais NL, Teschendorff AE, Green AR, Ellis IO (2007). MicroRNA expression profiling of human breast cancer identifies new markers of tumor subtype. Genome Biol.

[CR4] Chaffer CL, Weinberg RA (2011). A perspective on cancer cell metastasis. Science.

[CR5] Fang X, Cai Y, Liu J, Wang Z, Wu Q, Zhang Z, Yang CJ, Yuan L, Ouyang G (2011). Twist2 contributes to breast cancer progression by promoting an epithelial-mesenchymal transition and cancer stem-like cell self-renewal. Oncogene.

[CR6] Hong W, Dong E (2014). The past, present and future of breast cancer research in China. Cancer Lett.

[CR7] Ibrahim AF, Weirauch U, Thomas M, Grunweller A, Hartmann RK, Aigner A (2011). MicroRNA replacement therapy for miR-145 and miR-33a is efficacious in a model of colon carcinoma. Cancer Res.

[CR8] Li ZJ, Ou-Yang PH, Han XP (2014). Profibrotic effect of miR-33a with Akt activation in hepatic stellate cells. Cell Signal.

[CR9] Liu AY, Cai Y, Mao Y, Lin Y, Zheng H, Wu T, Huang Y, Fang X, Lin S, Feng Q (2014). Twist2 promotes self-renewal of liver cancer stem-like cells by regulating CD24. Carcinogenesis.

[CR10] Lu S, Azada MC, Ou SH (2015). Choroidal metastasis response to crizotinib in a ROS1-rearranged NSCLC patient. Lung Cancer.

[CR11] Marquart TJ, Allen RM, Ory DS, Baldan A (2010). miR-33 links SREBP-2 induction to repression of sterol transporters. Proc Natl Acad Sci USA.

[CR12] Micocci KC, Martin AC, Montenegro Cde F, Durante AC, Pouliot N, Cominetti MR, Selistre-de-Araujo HS (2013). ADAM9 silencing inhibits breast tumor cell invasion in vitro. Biochimie.

[CR13] Najafi-Shoushtari SH, Kristo F, Li Y, Shioda T, Cohen DE, Gerszten RE, Naar AM (2010). MicroRNA-33 and the SREBP host genes cooperate to control cholesterol homeostasis. Science.

[CR14] O’Shea C, McKie N, Buggy Y, Duggan C, Hill AD, McDermott E, O’Higgins N, Duffy MJ (2003). Expression of ADAM-9 mRNA and protein in human breast cancer. Int J Cancer (Journal international du cancer).

[CR15] Rayner KJ, Suarez Y, Davalos A, Parathath S, Fitzgerald ML, Tamehiro N, Fisher EA, Moore KJ, Fernandez-Hernando C (2010). MiR-33 contributes to the regulation of cholesterol homeostasis. Science.

[CR16] Rayner KJ, Esau CC, Hussain FN, McDaniel AL, Marshall SM, van Gils JM, Ray TD, Sheedy FJ, Goedeke L, Liu X (2011). Inhibition of miR-33a/b in non-human primates raises plasma HDL and lowers VLDL triglycerides. Nature.

[CR17] Rocks N, Paulissen G, El Hour M, Quesada F, Crahay C, Gueders M, Foidart JM, Noel A, Cataldo D (2008). Emerging roles of ADAM and ADAMTS metalloproteinases in cancer. Biochimie.

[CR18] Ruan K, Fang X, Ouyang G (2009). MicroRNAs: novel regulators in the hallmarks of human cancer. Cancer Lett.

[CR19] Shaw AT, Ou SH, Bang YJ, Camidge DR, Solomon BJ, Salgia R, Riely GJ, Varella-Garcia M, Shapiro GI, Costa DB (2014). Crizotinib in ROS1-rearranged non-small-cell lung cancer. N Engl J Med.

[CR20] Thomas M, Lange-Grunweller K, Weirauch U, Gutsch D, Aigner A, Grunweller A, Hartmann RK (2012). The proto-oncogene Pim-1 is a target of miR-33a. Oncogene.

[CR21] Volinia S, Galasso M, Sana ME, Wise TF, Palatini J, Huebner K, Croce CM (2012). Breast cancer signatures for invasiveness and prognosis defined by deep sequencing of microRNA. Proc Natl Acad Sci USA.

[CR22] Wan L, Pantel K, Kang Y (2013). Tumor metastasis: moving new biological insights into the clinic. Nat Med.

[CR23] Wang Z, Ouyang G (2012). Periostin: a bridge between cancer stem cells and their metastatic niche. Cell Stem Cell.

[CR24] Wang H, Sun T, Hu J, Zhang R, Rao Y, Wang S, Chen R, McLendon RE, Friedman AH, Keir ST (2014). miR-33a promotes glioma-initiating cell self-renewal via PKA and NOTCH pathways. J Clin Investig.

[CR25] Zheng L, Baumann U, Reymond JL (2004). An efficient one-step site-directed and site-saturation mutagenesis protocol. Nucleic Acids Res.

[CR26] Zhou Y, Huang Z, Wu S, Zang X, Liu M, Shi J (2014). miR-33a is up-regulated in chemoresistant osteosarcoma and promotes osteosarcoma cell resistance to cisplatin by down-regulating TWIST. J Exp Clin Cancer Res.

